# A Rare Cause of Neck Swelling: A Case Report and Review of Primary Pyomyositis of the Sternocleidomastoid

**DOI:** 10.7759/cureus.103500

**Published:** 2026-02-12

**Authors:** Daniel J Shepherd, Katerina Bopota, Wanyun Su, Andrew Barrett, Farzad Borumandi

**Affiliations:** 1 Oral and Maxillofacial Surgery, University Hospitals Sussex NHS Foundation Trust, Chichester, GBR; 2 Oral and Maxillofacial Surgery, Royal Surrey County Hospital, Guildford, GBR; 3 Histopathology, University Hospitals Sussex NHS Foundation Trust, Chichester, GBR

**Keywords:** case report, immunocompetent, neck abscess, primary pyomyositis, pyogenic myositis, review, sternocleidomastoid muscle (scm)

## Abstract

Primary pyomyositis is a rare bacterial infection of skeletal muscle occurring without a contiguous source of infection. Involvement of the sternocleidomastoid muscle (SCM) is exceptionally uncommon in temperate regions and may result in serious complications if diagnosis is delayed. We report a case of primary pyomyositis of the left SCM in a previously healthy 19-year-old male who presented with rapidly progressive neck pain and swelling nine days after minor blunt neck trauma. Contrast-enhanced computed tomography demonstrated an intramuscular SCM abscess with marked muscle oedema. Due to worsening pain and concern for evolving compartment syndrome, urgent surgical drainage and fasciotomy were performed, followed by delayed primary closure using a vessel-loop technique. The patient made a full functional recovery following antimicrobial therapy. To place this presentation in the clinical context, previously reported cases of primary SCM pyomyositis were examined. These reports highlight a consistent pattern of acute presentation, frequent diagnostic delay, and the potential for serious complications, including mediastinitis, when treatment is delayed. This case emphasises the importance of early imaging and timely surgical intervention in young, immunocompetent patients presenting with unexplained lateral neck swelling.

## Introduction

Primary pyomyositis is a rare primary bacterial infection of skeletal muscle that arises in the absence of any contiguous skin, soft tissue, or bone infection [1]. Secondary pyomyositis is much more common and simply refers to muscle involvement as a direct extension from a nearby infected site [1,2]. Primary pyomyositis usually involves large muscle groups such as the quadriceps, iliopsoas, or gluteals. Involvement of a small cervical muscle, such as the sternocleidomastoid muscle (SCM), is exceedingly rare [1,2]. Awareness of this rare condition is important as, without any obvious regional source, diagnosis is often delayed. When occurring in the neck, severe complications can result, including descending mediastinitis, internal jugular vein thrombosis, compartment syndrome, and systemic sepsis [1-4].

The exact pathogenesis of primary pyomyositis is not fully understood, as healthy muscle is generally considered resistant to bacterial invasion [4,5]. It is hypothesised that transient bacteraemia allows seeding of bacteria to skeletal muscles where an intra-muscular infective process occurs, especially in the presence of local and systemic factors that allow for bacterial proliferation. Primary pyomyositis is well recognised in the tropics, typically affecting healthy individuals under the age of 20. It accounts for as much as 2% of all paediatric admissions in some regions, and up to 25% of cases occur in otherwise healthy individuals (often preceded by minor trauma or vigorous exercise [1,2,4,6]. In contrast, it is seldom encountered in temperate climates such as the United Kingdom (UK), where it is classically thought to occur in patients with underlying systemic immunosuppression such as diabetes, HIV, or chronic corticosteroid use [1,2,4].

Primary pyomyositis, if untreated, classically progresses through three distinct clinical stages: an initial local diffuse inflammatory stage; a local suppurative or abscess stage; and finally, to a systemic or septic stage [5,7]. Early recognition and intervention during the initial or suppurative stages are critical to prevent progression to life-threatening complications [1-4,6].

The most recent review of primary pyomyositis of this topic is limited in scope and predates several subsequent reports [2]. We therefore present a case of primary SCM pyomyositis in an immunocompetent young adult in the United Kingdom, highlighting the diagnostic challenges associated with this rare presentation. To place this case in context and better define the spectrum of disease, we also performed a review of all reported cases of primary SCM pyomyositis in the literature, characterising patient risk factors, clinical presentation, management strategies, and outcomes in accordance with PRISMA 2020 guidelines [8].

## Case presentation

A 19-year-old previously healthy male rugby player presented with a two-day history of progressive left-sided neck pain and swelling, accompanied by rigors and malaise. Nine days prior to presentation, he had sustained minor blunt trauma to the left side of his neck while wrestling, with no immediate post-injury symptoms. He had no underlying medical conditions or relevant medical history.

On examination, the patient was febrile and tachycardic. The left side of the neck was markedly swollen, firm, and tender, extending from the mastoid region to approximately cervical levels II-III, with overlying erythema and severe restriction of neck movement. No alternative source of infection was identified on head and neck or systemic examination.

**Figure 1:**
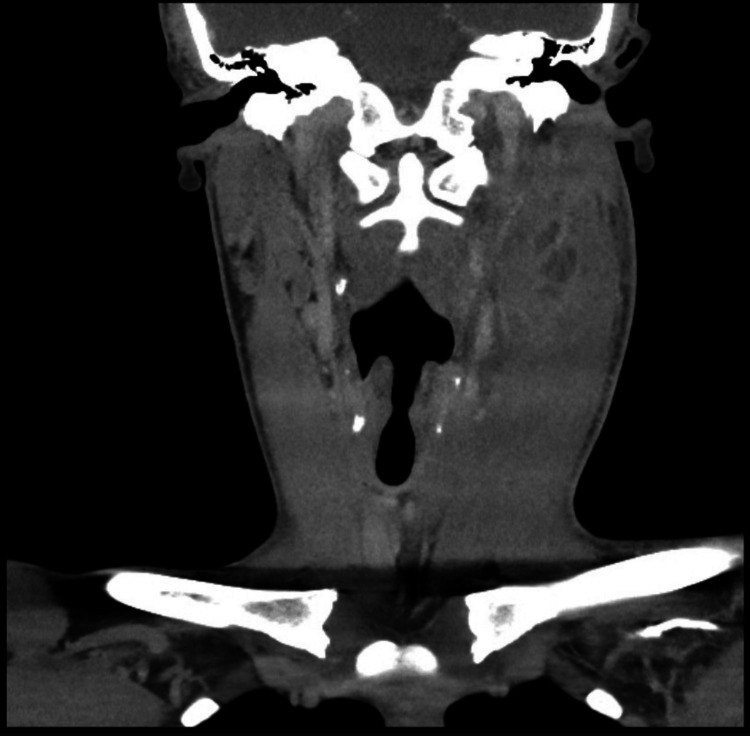
Coronal view of contrast-enhanced CT of the neck of a 19-year-old male immunocompetent athlete, demonstrating intra-muscular collection within the left SCM in the absence of local or regional source. SCM: Sternocleidomastoid muscle

Laboratory investigations on presentation demonstrated a pronounced inflammatory response, with markedly elevated C-reactive protein, leukocytosis, and neutrophilia (Table 1), while muscle enzymes, including creatine kinase and lactate dehydrogenase, were within normal reference ranges. HIV testing and blood glucose screening were unremarkable. Contrast-enhanced computed tomography of the neck revealed a 3.9 × 2.7 cm rim-enhancing intramuscular collection within the medial aspect of the left sternocleidomastoid muscle at level II, with diffuse muscle oedema and associated reactive lymphadenopathy (Figure 1). No local or regional source for the intramuscular infection was identified.

**Table 1:** Serial inflammatory markers at presentation, post-operatively, and follow-up.

	Day 0 (presentation)	Post-operative day 2	Post-operative day 10 (discharge)	Six-week follow-up	Laboratory Reference Range
C-Reactive Protein	197 mg/L (H)	50 mg/L (H)	3 mg/L	<1 mg/L	< 5 mg/L
Total White Blood Cell Count	15.5 × 10^9/L (H)	20.3 × 10^9/L (H)	9.9 × 10^9/L	5.3 × 10^9/L	4 – 10 × 10^9/L
Neutrophil Count	12.2 × 10^9/L (H)	18.6× 10^9/L (H)	7.1 × 10^9/L	2.4 × 10^9/L	2 – 7 × 10^9/L

Empirical intravenous co-amoxiclav was commenced on admission. Despite 24 hours of antimicrobial therapy, the patient developed worsening neck pain and increasing pressure that was disproportionate to examination findings, while inflammatory markers remained elevated (Table 1), raising concern for evolving compartment syndrome. Urgent surgical drainage and fasciotomy were therefore undertaken via a linear cervical incision.

Intraoperatively, the SCM was firm and tense, with gross oedema. Fasciotomy along the lateral aspect revealed markedly inflamed muscle fibres that were visibly separated and friable, consistent with intramuscular necrosis. Copious purulent material was drained from a cleft cavity on the medial aspect of the muscle at level II. Pus and tissue samples were obtained for microbiological and histopathological analysis. The carotid sheath was mobilised from the medial SCM fascia, with both the internal jugular vein and carotid artery found to be patent.

**Figure 2:**
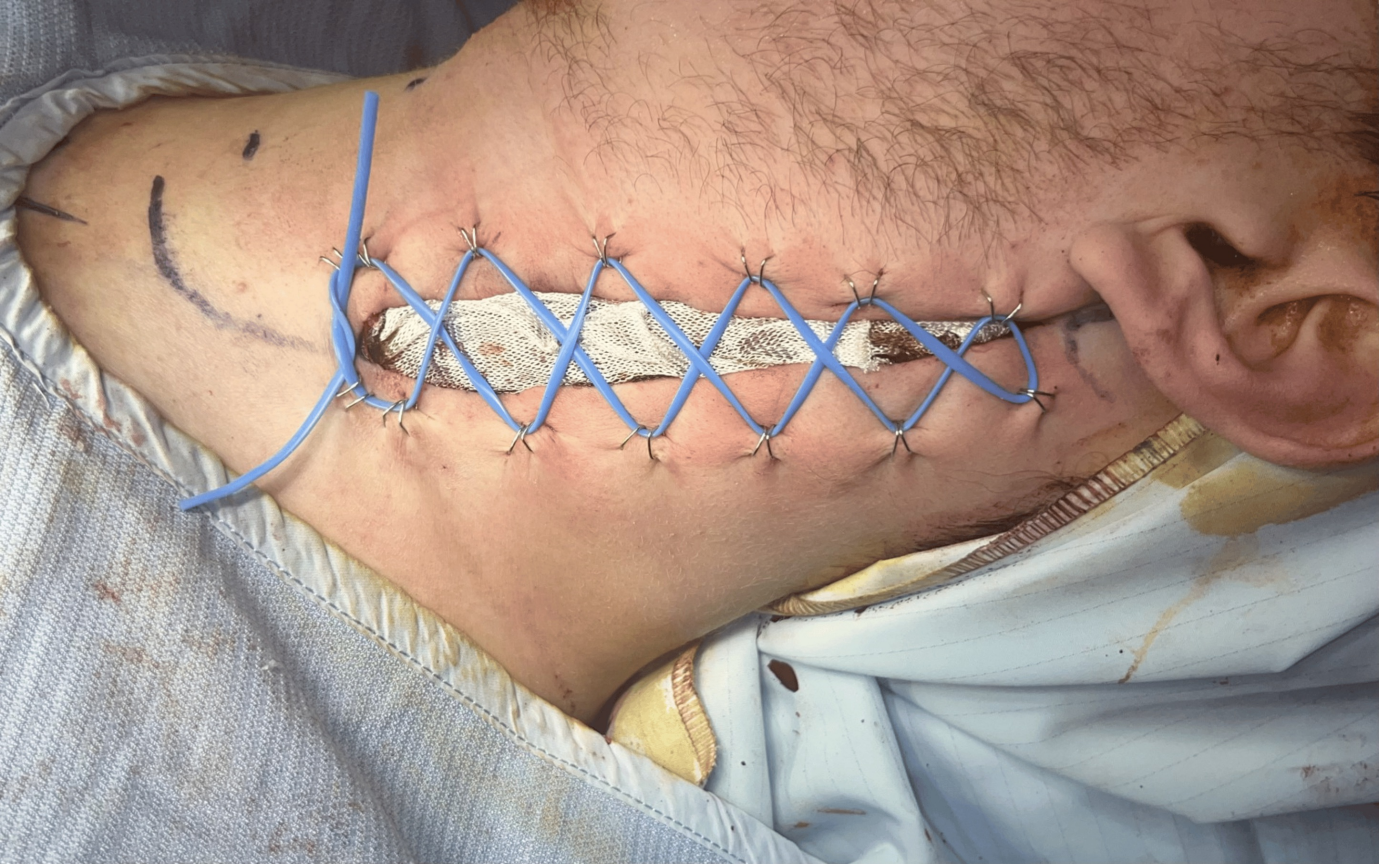
Left neck wound closure using the vessel loop (“shoelace”) technique due to tense muscle oedema.

Given the degree of muscle oedema and concern for sustained compartment pressure, delayed primary closure was performed using a vessel-loop “shoelace” technique to allow gradual approximation of the skin edges as swelling subsided (Figure 2), with definitive closure achieved on post-operative day five. Inflammatory markers improved rapidly following surgical intervention and normalised by the time of discharge on post-operative day 10, remaining within normal limits at six-week follow-up (Table 1).

**Figure 3:**
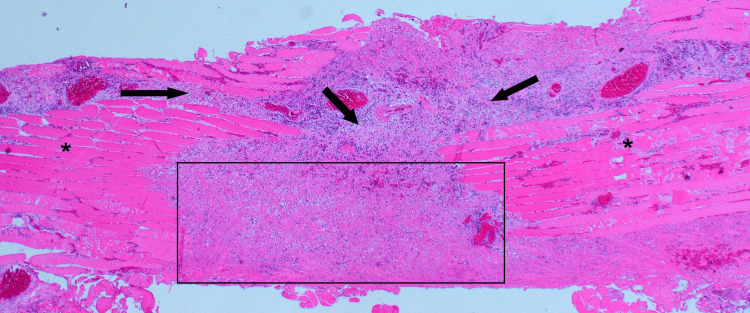
Bundles of striated muscle (asterisks) disrupted by infiltrates of acute and chronic inflammatory cells (arrows) leading to localised muscle necrosis (rectangle). H&E, original magnification x 20.

Histological examination confirmed features consistent with pyomyositis (Figure 3). Blood and intraoperative cultures were negative. The patient completed a 14-day course of co-amoxiclav. Follow-up ultrasound demonstrated complete resolution of the abscess, and at six-week review, the wound had healed fully with restoration of normal neck function.

## Discussion

**Figure 4:**
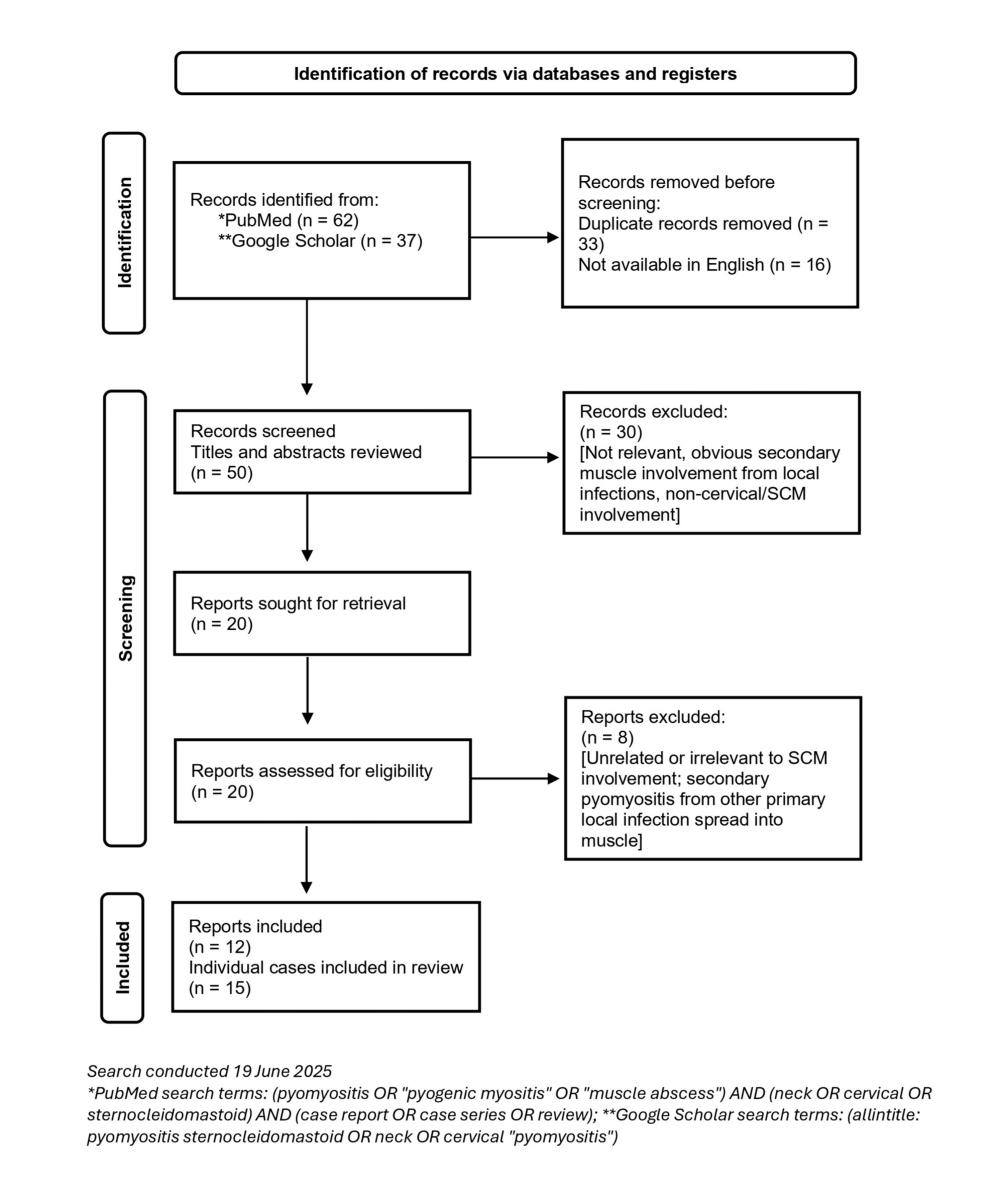
Flow diagram for review of the literature for sternocleidomastoid pyomyositis.

Primary pyomyositis of the SCM presenting in a young, immunocompetent patient is an exceptionally rare diagnosis in temperate regions such as the UK. In this case, minor blunt neck trauma was followed by rapidly progressive pain and swelling, highlighting the diagnostic and management challenges associated with this uncommon presentation. To place these findings in context, previously reported cases of primary SCM pyomyositis were identified through a structured review of the literature (strategy outlined in Figure 4). The key clinical features, management strategies, and outcomes from previously reported cases are summarised in Table 2, allowing direct comparison with the presentation and clinical course observed in our patient.

**Table 2:** Review of literature of the reported cases of primary pyomyositis of the sternocleidomastoid from 1992 to 2024. DM: Diabetes mellitus; CT: computed tomography; SCM: sternocleidomastoid; IV: intravenous; QDS: four times daily; US: ultrasound; ROM: range of motion; TDS: three times daily. Source indexing: * indicates report indexed in MEDLINE; ** indicates non-MEDLINE indexed journal or abstract; # indicates conference abstract or non-indexed source.

	Age/Sex	Co-morbidities/Risk Factors	Clinical Presentation	Imaging	Microbiology	Management	Complications	Outcome
*Gorospe et al., 2017 [1]	21 Male	None	Neck pain; tender mass; fever	CT: SCM abscess with descending mediastinitis	Group A Streptococcus	Surgical drainage of SCM & mediastinum; IV antibiotics	Mediastinitis	Full recovery
*Gosnell et al., 2016 [2]	48 Male	None	1-week neck pain & swelling; fever; limited ROM	CT: abscess in SCM	Group A Streptococcus	Surgical drainage; IV co-amoxiclav (1.2g TDS) switched to benzylpenicillin (1.2g QDS x 4 days) then oral clindamycin (450mg QDS x 4 weeks)	None reported	Full recovery
**Soleh & Mohamad, 2010 [3]	72 Female	Poorly controlled type II DM	1-week neck swelling, pain, fever	CT: abscess in SCM; parotid gland displaced	Klebsiella pneumoniae	Surgical drainage; IV co-amoxiclav and metronidazole	None reported	Full recovery; diabetes improved
*Collier et al., 2010 – Case 1 [6]	Young adult Female	Recent travel to Africa (tropical region)	Fever, neck pain, no trauma	US/CT: diffuse SCM swelling, no abscess	No organism isolated	IV flucloxacillin + benzylpenicillin (2.4g QDS); no surgery (doubled dose of antibiotics)	None reported	Full recovery once the antibiotic dose increased
*Collier et al., 2010 – Case 2 [6]	Young adult Female	Systemic Lupus Erythematosus (prednisolone); recent travel to Africa (tropical region)	Fever, neck pain, no trauma	US/CT: diffuse SCM inflammation, no abscess	Group A Streptococcus	IV flucloxacillin + benzylpenicillin; no surgery (doubled dose of antibiotics)	None reported	Full recovery once the antibiotic dose increased
**Snyder et al., 2018 [9]	22 Male	Blunt trauma to the neck 3 months prior	3-month history of neck pain & swelling, weight loss, fevers, reduced ROM	MRI: SCM mass with medial spread to deep neck spaces	Staphylococcus epidermidis & Propionibacterium acnes	Surgical drainage, IV antibiotics, then oral (not specified)	None reported	Full recovery reported
*Rimell et al., 1992 [10]	43 Male	Rheumatoid Arthritis (prednisone + azathioprine); Type II DM	Neck pain & swelling following joint infection, progression to sepsis	CT: bilateral abscesses in SCM & mediastinal spread	Staphylococcus aureus	Multiple surgical debridements of neck & infected joints; IV vancomycin	Multiple arthrotomies of other infected joints	Recovered after a prolonged 12-week inpatient stay
*López-Rodríguez et al., 2008 [11]	46 Male	None	7 days of difficulty elevating the arm, erythematous induration over the sternal manubrium	CT: abscesses in distal SCM & pectoralis major	Staphylococcus aureus	IV Cloxacillin (1 g QDS x 10 days) then oral Cloxacillin (1g QDS x 8 weeks)	None reported	Recovered (5 months of follow-up)
*Sakaida et al., 2016 [12]	62 Male	Poorly controlled type II diabetes mellitus (HbA1c 12.1%)	1-week neck swelling, fever	US: swollen SCM with hypoechoic mass; CT: abscess in SCM	Streptococcus anginosus	US-guided aspiration + surgical drainage; IV antibiotics	None reported	Full recovery; discharged on day 16
#Haroun et al., 2022 (Abstract only) [13]	55 Male	Liver cirrhosis; uncontrolled DM	6-day neck swelling, no fever	CT: SCM abscess & early mediastinitis	Salmonella enterica	Surgical drainage; IV ceftriaxone changed to oral trimethoprim-sulfamethoxazole	Mediastinitis	Recovery; residual induration at 4 weeks
*Casillas et al., 2023 [14]	61 Male	Type II DM (HbA1c 8.5%)	9 days of right neck pain/swelling, initially afebrile, later febrile	Initial CT: swelling only; repeat CT: abscess in SCM	Staphylococcus aureus	Initial oral doxycycline; later IV therapy + surgical drainage; 6-week daptomycin	None reported	Improved; discharged on antibiotics
*Fraser et al., 2024 (Abstract only) [15]	Elderly Male	None	7 days of worsening neck pain, swelling, fever	CT: collection within SCM	Not available	Surgical drainage & debridement; 14 days IV antibiotics	None reported	Recovery implied; followed up in clinic
**Neshat et al., 2024 – Case 3 [16]	41 Male	DM (unspecified)	Bilateral neck swelling, pain	CT: bilateral bulky SCM with peripheral enhancement and air foci	Klebsiella pneumoniae	Surgical drainage; cephalosporins, carbapenems (unclear dose/duration)	Delayed wound closure	Improved within 2 months
**Neshat et al., 2024 – Case 6 [16]	45 Male	DM (unspecified)	Bilateral neck swelling, dyspnea & dysphagia	CT: right SCM abscess, pectoralis major abscess, mediastinal spread	Bacteroides fragilis	Surgical drainage. Antibiotics (unclear antibiotic choice & dose/duration)	Mediastinitis	Recovery implied
**Neshat et al., 2024 – Case 7 [16]	48 Male	None	1 week of neck pain, swelling, fever	CT: abscess in SCM; no mediastinal spread	Staphylococcus aureus	Surgical drainage; IV Co-amoxiclav (1.2g TDS) then benzylpenicillin (1.2g QDS x4 days), oral clindamycin (450mg QDS x 4 weeks)	None reported	Full recovery; no residual infection

The literature review identified only 15 reported cases of primary SCM pyomyositis over the last 33 years, with just seven occurring in patients without identifiable immunosuppression (Table 2). This contrasts with the traditional perception of primary pyomyositis as a disease predominantly affecting immunocompromised individuals. Notably, only one previously reported case described primary SCM pyomyositis in an immunocompetent young adult following minor blunt neck trauma, mirroring the presentation observed in our patient [9].

Unlike primary pyomyositis affecting other muscle groups, diabetes mellitus, rather than haematological malignancy or HIV infection, appears to be the most frequently associated comorbidity. The condition also demonstrates a marked male predominance, with 12 of the 15 reported cases occurring in male patients, a finding that may relate to relative skeletal muscle mass. While SCM involvement remains rare in the literature, data from tropical regions suggest that healthy individuals with a history of trauma or strenuous physical activity represent a recognised subset of primary pyomyositis affecting other muscle groups [4-6].

Reported cases of primary SCM pyomyositis demonstrated a remarkably consistent clinical presentation, characterised by the rapid onset of painful lateral neck swelling, usually accompanied by fever and elevated inflammatory markers. Similar to our patient, several reports described pain that appeared disproportionate to early examination findings, which may represent an important diagnostic clue [1-4,6,7,14,16]. Nearly all cases presented in the suppurative stage, with an established intramuscular abscess within the SCM at the time of diagnosis. Early misdiagnosis was common, reflecting the unusual occurrence of an isolated intramuscular neck abscess in the absence of a local or regional source. Initial alternative diagnoses included parotid abscess, cellulitis, lymphadenitis, and neoplastic processes, with diagnostic delay in some cases resulting in serious complications, most notably descending mediastinitis [3,9].

Given the potentially life-threatening sequelae of deep neck infections, imaging was frequently guided by availability in the acute setting. Cross-sectional imaging played a central role in confirming the diagnosis and informing surgical planning. Contrast-enhanced CT was the most commonly utilised modality, consistently demonstrating rim-enhancing intramuscular collections with surrounding muscle oedema [1-3,12]. Although MRI is more sensitive for detecting early myositis, it was rarely used in reported cases of SCM pyomyositis, likely due to clinical urgency and resource constraints. Ultrasound was occasionally employed for initial assessment or image-guided aspiration but was insufficient as a sole imaging modality in cases with deep or extensive disease [12].

Once suppuration was confirmed, most patients underwent open surgical drainage, typically via transverse or longitudinal cervical incisions [1-3,7,9,10,12-16]. A small number of early cases without a discrete abscess were successfully managed with intravenous antibiotics alone [6,11]. Several cases were complicated by mediastinitis, reinforcing the importance of early diagnosis and timely intervention. In our patient, progressive pain, tense swelling, and concern for evolving compartment physiology prompted urgent surgical decompression. To our knowledge, compartment syndrome of the SCM has not previously been described. Decompression was achieved via longitudinal fasciotomy with delayed primary closure using a vessel-loop “shoelace” technique, a method commonly employed in limb fasciotomies but not previously reported in the management of SCM pyomyositis (Figure 2).

Microbiological findings across reported cases mirrored those seen in pyomyositis affecting other muscle groups, with staphylococcal and streptococcal species predominating [17-19]. Empirical antibiotic regimens generally targeted these organisms, most commonly using penicillin-based therapies, and several reports noted clinical improvement only after escalation or increased dosing [2,6,16]. Once culture results were available, antimicrobial therapy was adjusted and continued for a total duration of two to six weeks, depending on disease severity. Culture-negative cases, including ours, were not uncommon and likely reflect prior antibiotic exposure in the setting of a sensitive causative organism. The isolation of atypical pathogens in a minority of cases further emphasises the importance of microbiological sampling to guide targeted therapy.

When recognised and managed promptly, outcomes were favourable across all reported cases. In contrast, diagnostic delay was associated with significant morbidity, including descending mediastinitis, internal jugular vein thrombosis, and sepsis [1-6,10,13,16]. Despite these complications, no mortality has been reported. In our case, early imaging, timely surgical decompression, and appropriate antimicrobial therapy resulted in full functional recovery, consistent with outcomes reported in cases managed without delay.

## Conclusions

Primary pyomyositis of the SCM, although rare, should be considered in the differential diagnosis of neck pain with swelling and fever, even in a young, immunocompetent patient. It is a common misconception that only immunocompromised patients are at risk for this condition, especially in temperate regions, such as the UK. This case and our literature review emphasise the diagnostic and therapeutic challenges of primary pyomyositis of the SCM. A high index of suspicion, early imaging and appropriate surgical and antimicrobial treatment are key to reducing morbidity and have been associated with favourable outcomes in these unusual and rare cases.

## References

[1] Gorospe Sarasúa L, Valdebenito-Montecino AP, Muñoz-Molina GM (2017). Descending necrotizing mediastinitis secondary to spontaneous sternocleidomastoid muscle abscess. Arch Bronconeumol.

[2] Gosnell EJ, Anwar B, Varadarajan V, Freeman S (2016). Sternocleidomastoid pyomyositis. Eur Ann Otorhinolaryngol Head Neck Dis.

[3] Soleh MN, Mohamad I (2010). Sternocleidomastoid pyomyositis mimicking parotid abscess. Bangladesh J Med Sci.

[4] Bickels J, Ben-Sira L, Kessler A, Wientroub S (2002). Primary pyomyositis. J Bone Joint Surg Am.

[5] Chauhan S, Jain S, Varma S, Chauhan SS (2004). Tropical pyomyositis (myositis tropicans): current perspective. Postgrad Med J.

[6] Collier S, Vig N, Collier J (2010). Two cases of tropical pyomyositis of the sternocleidomastoid muscle occurring in the UK. Br J Oral Maxillofac Surg.

[7] Flier S, Dolgin SE, Saphir RL, Shlasko E, Midulla P (2003). A case confirming the progressive stages of pyomyositis. J Pediatr Surg.

[8] Page MJ, Moher D, Bossuyt PM (2021). PRISMA 2020 explanation and elaboration: updated guidance and exemplars for reporting systematic reviews. BMJ.

[9] Snyder V, Le T, Wood V (2018). Atypical presentation of pyomyositis in a young immunocompetent male with prior neck trauma: a case report. J Otolaryngol ENT Res.

[10] Rimell F, Dohar J, Niehans G, Goding GS (1992). Cervical "tropical" pyomyositis. Otolaryngol Head Neck Surg.

[11] López-Rodríguez R, Campos-Franco J, Mallo-González N, Alende-Sixto MR, González-Quintela A (2008). Sternocleidomastoid and pectoralis major pyomyositis in an immunocompetent patient. Orthopedics.

[12] Sakaida H, Matsuda Y, Takeuchi K (2017). Sonographic appearance of pyomyositis of the sternocleidomastoid muscle: a case report. J Clin Ultrasound.

[13] Haroun P, Yang S, Adamson PC, Sakona AN (2022). 318. A pain in the neck: cervical pyomyositis, a rare case of extraintestinal nontyphoidal salmonellosis further characterized by whole-genome sequencing. Open Forum Infect Dis.

[14] Casillas-Berumen SS, Kasanga S, Salik A (2023). Does that go there? A rare occurrence of spontaneous sternocleidomastoid abscess without trauma in a diabetic individual. Am J Case Rep.

[15] Fraser C, Thompson CS, Yeo JC (2024). Isolated left sternocleidomastoid pyomyositis: a rare presentation of cervical sepsis. BMJ Case Rep.

[16] Neshat H, Wahid S, Reddy MM, Priyanka D (2024). Pyomyositis neck as a diagnostic challenge in tropics - a case series. Int J Acad Med Pharm.

[17] Shittu A, Deinhardt-Emmer S, Vas Nunes J, Niemann S, Grobusch MP, Schaumburg F (2020). Tropical pyomyositis: an update. Trop Med Int Health.

[18] Chiedozi LC (19791). Pyomyositis: review of 205 cases in 112 patients. Am J Surg.

[19] Comegna L, Guidone PI, Prezioso G (2016). Pyomyositis is not only a tropical pathology: a case series. J Med Case Rep.

